# Extra-Anatomical Bypass Grafting and Latissimus Dorsi Myocutaneous Flap Reconstruction for Post-sternotomy Mediastinitis With Prosthetic Aortic Graft Infection

**DOI:** 10.7759/cureus.18086

**Published:** 2021-09-18

**Authors:** Daiki Kitano, Jiro Matsuo, Shunsuke Sakakibara, Atsushi Oomura, Takeo Osaki, Kenji Okada, Hiroto Terashi

**Affiliations:** 1 Plastic Surgery, Kobe University, Kobe, JPN; 2 Department of Cardiovascular Surgery, Kobe University Graduate School of Medicine, Kobe, JPN; 3 Department of Plastic Surgery, Kobe University Graduate School of Medicine, Kobe, JPN; 4 Plastic Surgery, Hyogo Cancer Center, Akashi, JPN

**Keywords:** pedicled latissimus dorsi myocutaneous flap, negative pressure wound therapy with continuous irrigation, extra-anatomical bypass grafting, aortic arch graft infection, post-sternotomy mediastinitis

## Abstract

Extra-anatomical bypass grafting is a surgical method used to remove an infected aortic graft and promote revascularization with a new graft in the non-infected area. Here, we report a case of intractable post-sternotomy mediastinitis (PSM) with aortic graft infection which was treated with extra-anatomical bypass grafting. A 56-year-old woman with a history of multiple aortic dissection and prosthetic graft replacement in the thoracoabdominal area developed PSM with aortic arch graft infection. Bacterial culture of the exposed prosthetic graft tissue yielded multidrug-resistant *Pseudomonas aeruginosa*. Meticulous debridement of the wound and management by negative pressure wound therapy with continuous irrigation was performed. However, the infection of the prosthetic graft could not be controlled. Extra-anatomical bypass was performed between the left common carotid artery and right subclavian artery via the right common carotid artery. Then, the infected graft was removed. After the resolution of infection, the mediastinal wound was reconstructed with a pedicled latissimus dorsi myocutaneous flap, which was harvested from the right dorsum. No recurrence of infection occurred in the nine-month follow-up period. Debridement and removal of exposed artificial graft are considered the gold standard for treating wound infection. In situ replacement of infected aortic grafts carries a risk of re-infection due to residual bacterial contamination of the periprosthetic tissue. Extra-anatomical bypass would be a useful option for reducing the risk of re-infection in patients with intractable PSM and prosthetic aortic graft infection. Further studies are warranted to evaluate the risks and benefits of this operative method.

## Introduction

Post-sternotomy mediastinitis (PSM) is one of the most serious complications that can arise after open heart surgery [1]. Infection of deep sternal organs, including the heart, great vessels, and lungs, leads to a severe clinical course and potentially death. Debridement of necrotic tissues and wound lavage are the first-line treatment for infection control. For patients with prolonged prosthetic graft infection, graft removal and replacement are considered. However,in situ graft replacement operations pose a large physical burden to the patient and are associated with a high risk of infection recurrence.

Extra-anatomical bypass grafting involves removal of the infected graft and revascularization using a new graft via a root away from the normal anatomy. In 1977, Brown et al. introduced this method for the treatment of infective endocarditis with prosthetic aortic valve infection [2]. This innovative method has been accepted as a last resort to cope with prosthetic graft infection. Here, we report a case of intractable PSM treated with extra-anatomical bypass grafting to remove the infected graft and prevent the recurrence of infection.

## Case presentation

**Figure 1:**
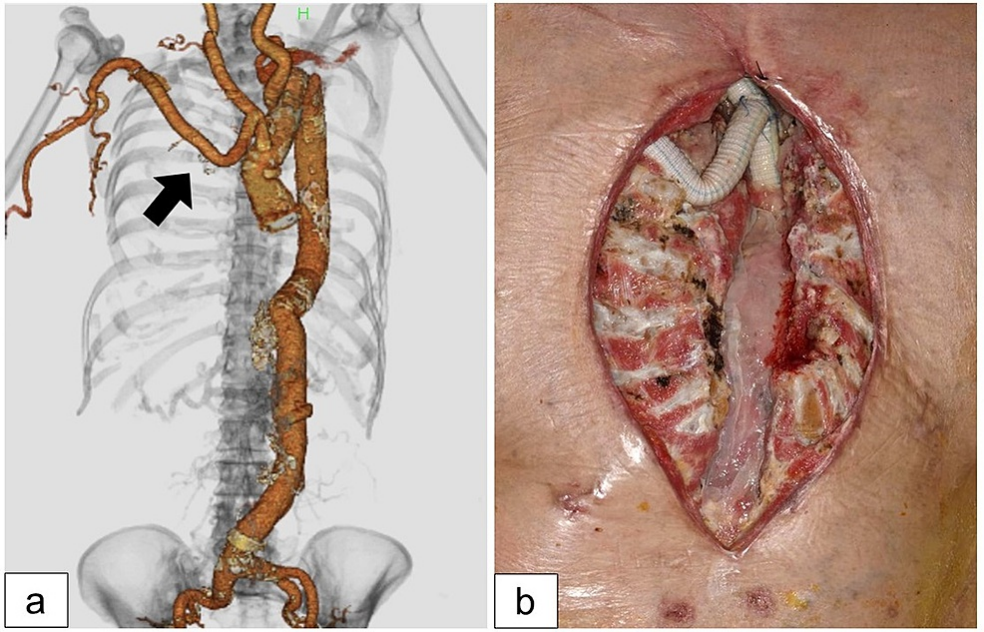
On admission. (a) Computed tomography angiography of the aorta. The arrow shows the replaced prosthetic graft during the last operation. (b) The infected prosthetic graft was exposed within the mediastinal wound without the sternal bone.

A 56-year-old woman with Marfanoid habitus underwent multiple prosthetic graft replacement operations for thoracoabdominal aortic dissection. Two months before admission to our hospital, she was diagnosed with an impending rupture of the brachiocephalic aneurysm. Removal of the aneurysm and prosthetic graft replacement via median sternotomy was performed at another hospital (Figure 1a). She developed PSM with prosthetic graft infection caused by multidrug-resistant *Pseudomonas aeruginosa*. Three weeks after the operation, re-sternotomy and debridement of the necrotic tissues were performed. However, she suffered from uncontrollable sepsis with multidrug-resistant *P. aeruginosa* and was transferred to our hospital.

At the initial presentation, we confirmed that the sternal bone was completely debrided, and the prosthetic graft was exposed to the mediastinal wound (Figure 1b). Bacterial culture of the periprosthetic tissues yielded multidrug-resistant *P. aeruginosa*. Pulsed wound lavage with 10 L of normal saline using PulsaVAC® (Zimmer Biomet Holdings, Inc., USA) was performed under general anesthesia. The wound was managed by negative pressure wound therapy with continuous irrigation (NPWTci) [3], which consisted of continuous normal saline irrigation at 100 mL/hour via three tubes and application of 75 mmHg of negative pressure via polyurethane foam.

The foam dressing change and wound lavage using PulsaVAC® were performed twice a week in the operating room. Thirty-five days after the initiation of NPWTci, healthy granulation tissue formation was confirmed in the wound. However, the wound culture results continued to be positive for *P. aeruginosa*. Removal of the infected prosthetic graft and re-replacement of the graft were considered to control the infection. However,in situ replacement of infected prosthetic grafts has a high risk of bacterial contamination as the periprosthetic infectious tissues can contaminate the new graft. Therefore, extra-anatomical bypass grafting has been proposed as an alternative treatment.

**Figure 2:**
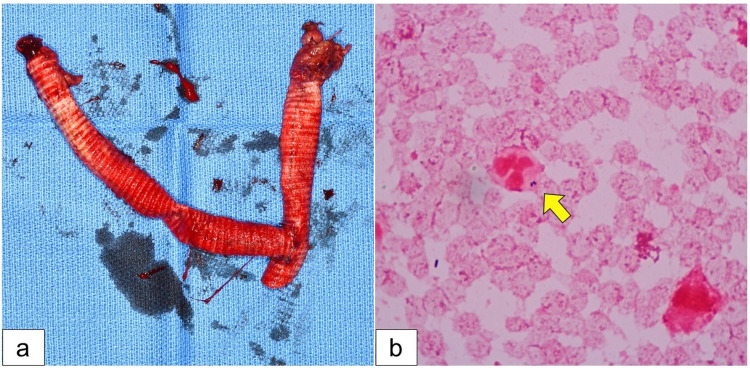
Removal of the infected graft. (a) The exposed prosthetic graft was removed. (b) Gram staining of the periprosthetic tissues confirmed phagocytosis of bacteria by neutrophils (arrow).

**Figure 3:**
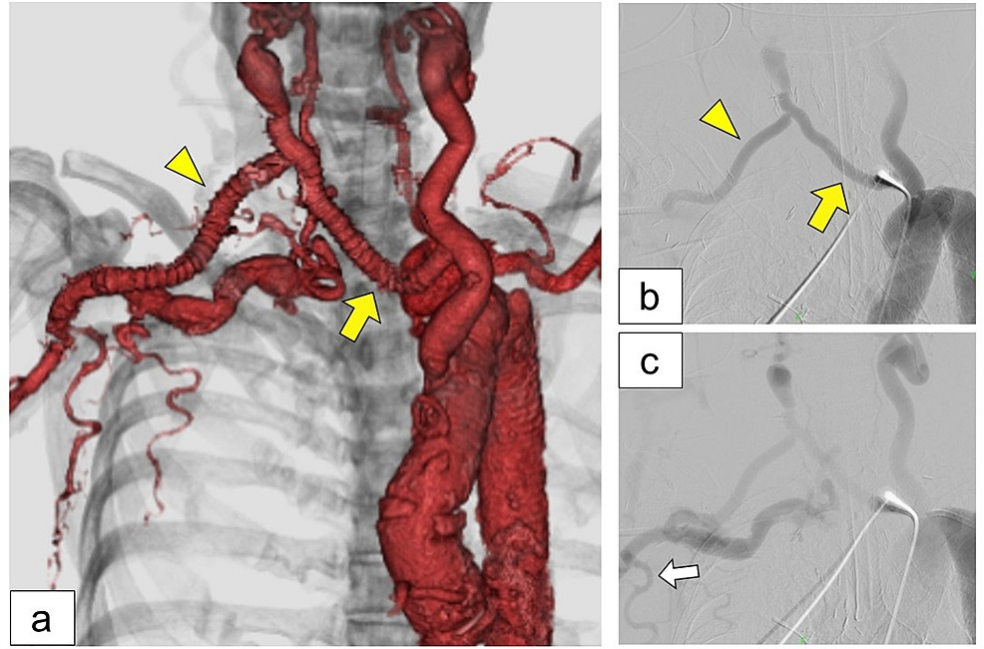
After the extra-anatomical bypass operation. (a) Postoperative computed tomography angiography demonstrated a new prosthetic graft that connected the left common carotid artery and right subclavicular artery (yellow arrowhead) via the right common carotid artery (yellow arrow). (b, c) Postoperative angiography of the ascending aorta revealed anterograde blood flow in the new graft (yellow arrow and arrowhead). The white arrow indicates the right thoracodorsal artery.

The infected graft, which was exposed to the mediastinal wound, was removed (Figure 2a). Microscopic examination of the infected prosthetic graft tissues revealed bacterial phagocytosis by neutrophils (Figure 2b). The new graft was placed to bypass between the left common carotid artery (CCA) and right subclavicular artery (SCA) via the right CCA (Figure 3a). At the time of the new bypass graft placement, we paid attention to prevent bacterial contamination from the mediastinal wound. Postoperative angiography revealed anterograde blood flow from the left CCA to the right SCA via the right CCA through the new graft, which was anastomosed to the left CCA (Figure 3b). Arterial blood circulation in the distal area of the right SCA, including the right thoracodorsal artery, was maintained (Figure 3c).

**Figure 4:**
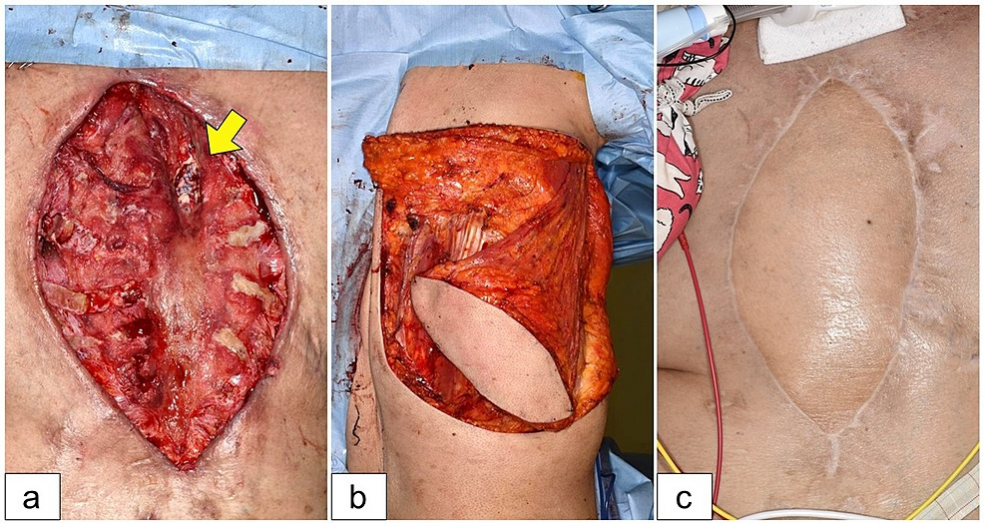
Latissimus dorsi myocutaneous flap reconstruction. (a) Healthy granulation tissue formation suggesting that the removal of the infected prosthetic graft promoted mediastinal wound healing. The arrow indicates the stump of the ascending aorta. (b) The sternal wound was reconstructed using a pedicled latissimus dorsi myocutaneous flap, which was perfused by the right thoracodorsal artery. (c) The flap survived completely. There were no signs of infection recurrence nine months postoperatively.

NPWTci was used for wound management until flap coverage. Red granulation tissue growth in the mediastinal wound was confirmed in the area of the infected graft was removed (Figure 4a). Seven days after the extra-anatomical bypass operation, the wound culture tested negative for *P. aeruginosa*. The pedicled latissimus dorsi myocutaneous flap, which was perfused by the right thoracodorsal artery, was elevated (Figure 4b). The flap was passed through a subcutaneous tunnel between the right pectoralis major muscle and the chest. The apex of the flap was placed within the mediastinum to fill the dead space. The flap survived completely with the elimination of inflammatory reactions. The patient was then transferred to a previous hospital. At nine months postoperatively, no signs of infection recurrence had occurred (Figure 4c).

## Discussion

The gold standard in the treatment of periprosthetic tissue infection is debridement and removal of the infected prosthesis. However, removal of the infected graft can be challenging in patients with prosthetic graft infection after cardiovascular surgery. High perioperative mortality has been reported after the removal and replacement of infected grafts. Furthermore, the procedure has a high physical burden on patients [4].

Therefore, local infection control and coverage with a well-vascularized flap are often attempted to avoid graft replacement [5]. NPWTci is a suitable management method for mediastinal wounds with prosthetic graft exposure. Ikeno et al. reported favorable outcomes of NPWTci in 18 patients with PSM and prosthetic graft infection [3]. NPWTci aims to control wound infection and minimize the chance of prosthetic graft infections recurring when a negative bacterial culture is obtained.

Despite these measures, replacement operations are still required in some patients with prolonged positive bacterial cultures of periprosthetic tissue.In situ replacement of the infected graft is associated with a high risk of infection of the new graft due to the persistence of infection in the surrounding tissue of the infected graft. Patients with re-infection of the replaced graft develop a severe clinical course, including septic shock and multiple organ failure. Therefore, extra-anatomical bypass grafting is presented as a last resort in cases of intractable graft infections [6].

Several researchers have reported the usefulness of removing the infected graft followed by revascularization with extra-anatomical bypass grafting in PSM patients with prolonged prosthetic graft infections [7,8]. Seeger et al. reported that the three-year survival rate after extra-anatomical bypass grafting was 81% and the re-infection rate was only 3%. However, the subjects of these two studies were patients with prosthetic graft infections after abdominal aortic surgery at renal artery levels or lower.

Limited studies have been conducted regarding extra-anatomical bypass for total arch graft infections. Inoue et al. reported a case of extra-anatomical bypass grafting for an infective aortic arch aneurysm [9]. The aneurysm and surrounding tissues were highly contaminated with purulent discharge. They decided to perform extra-anatomical bypass grafting from the ascending aorta to the descending aorta, rather than in situ replacement, to prevent re-infection of the graft. After the operation, the sternal wound was closed without flap reconstruction.

Although the combination of extra-anatomical bypass grafting and flap coverage could be an alternative treatment option for the PSM patients with total arch graft infection, not all patients were subjected to this aggressive treatment. We need to consider the indication of this treatment from the aspect of severe operative invasiveness and possible postoperative unfavorable result. In other words, hemodynamically unstable patients are not subjected to this aggressive surgery. The reason why we performed this surgery was that our patient had a capacity for surgical invasion.

Extra-anatomical bypass grafting causes hemodynamic changes in the distal bypass area. The latissimus dorsi myocutaneous flap is perfused by the thoracodorsal artery, which bifurcates from the axial artery. In our case, the right axial artery received blood supply from the left CCA via the bypass graft. Despite the circulation pathway change in the proximal area of the vascular pedicle, the flap completely survived without ischemia. The extra-anatomical bypass grafting did not interfere with reconstruction using the pedicled latissimus dorsi myocutaneous flap.

## Conclusions

We have reported a case of intractable PSM with a prosthetic aortic graft infection that was successfully managed by extra-anatomical bypass grafting. Instead of in situ replacement of the infected graft, extra-anatomical bypass offered a new route outside the infected wound. Extra-anatomical bypass grafting would be a useful option for patients at high risk of re-infection with in situ replacement of the infected graft. Further studies are warranted to evaluate the risks and benefits of this operative method.
